# Nanozyme Microrobots: Programmable Spatiotemporal Catalysis for Targeted Therapy and Diagnostics

**DOI:** 10.1002/advs.202523365

**Published:** 2026-01-28

**Authors:** Hong Huy Tran, Nil Kanatha Pandey, David P. Cormode, Daeyeon Lee, Edward Steager, Hyun Koo

**Affiliations:** ^1^ Department of Chemical and Biomolecular Engineering School of Engineering & Applied Science University of Pennsylvania Philadelphia Pennsylvania USA; ^2^ Department of Orthodontics and Divisions of Pediatric Dentistry and Community Oral Health School of Dental Medicine University of Pennsylvania Philadelphia Pennsylvania USA; ^3^ Center for Innovation & Precision Dentistry School of Dental Medicine School of Engineering and Applied Science University of Pennsylvania Philadelphia Pennsylvania USA; ^4^ Biofilm Research Laboratories Levy Center for Oral Health School of Dental Medicine University of Pennsylvania Philadelphia Pennsylvania USA; ^5^ Department of Radiology Perelman School of Medicine University of Pennsylvania Philadelphia Pennsylvania USA; ^6^ Department of Bioengineering School of Engineering & Applied Science University of Pennsylvania Philadelphia Pennsylvania USA; ^7^ Department of Mechanical Engineering and Applied Mechanics School of Engineering and Applied Science University of Pennsylvania Philadelphia Pennsylvania USA

**Keywords:** localized catalysis, biomedical robots, close‐loop feedback control, reactive oxygen species, stimuli‐responsive actuation, structure‐activity relationships

## Abstract

Nanozyme microrobots combine catalytic nanomaterials with small‐scale robotic control to deliver programmable, spatiotemporal catalysis for biomedical applications with precision. Actuated by external stimuli, such as magnetic, acoustic, optical, or chemical gradients, these systems localize and modulate catalytic activity on demand, overcoming long‐standing limitations of bulk catalysis, including poor spatial precision, restricted substrate access, and limited adaptability in complex biological environments. By uniting targeted navigation with stimulus‐responsive activation, nanozyme microrobots facilitate precise intervention in anatomically challenging and inaccessible niches, from biofilms to solid tumors, and support theranostic workflows with real‐time readouts. This review focuses on design principles for integrating nanozymes with microrobotics, surveys actuation, automation, and control strategies, and highlights biomedical applications across biofilm infection control, oncology, and catalytic diagnostics. Together, the convergence of nanozyme catalysis and microrobotic mobility is yielding versatile, adaptive platforms with the potential to transform targeted diagnostics and therapy.

## Graphical abstract

This review presents nanozyme microrobots as emerging catalytic systems that integrate mobility, external actuation, and adaptive reactivity to achieve precise biochemical functions. By examining mobility‐regulated catalysis, spatial targeting, integrated designs, and translational demonstrations, the article highlights how nanozyme robotics enables programmable therapeutic and diagnostic interventions in complex biological environments.

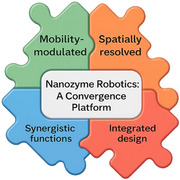

## 1 Introduction

Nanozymes are nanomaterials that mimic the catalytic functions of natural enzymes [1, 2, 3]. Compared to natural enzymes, nanozymes offer high stability, tunable activity, and compatibility with diverse environments [4, 5]. The materials base for nanozymes is diverse and reflects the broad chemical origins of catalytic activity. Transition metal oxides (Fe_3_O_4_, MnO_2_, Co_3_O_4_) remain prominent owing to multivalent redox centers and ease of synthesis [6]. Noble metals (Au, Pt, Pd) provide superior catalytic turnover and stability but are limited by high cost and, in some cases, poor biocompatibility [7]. Carbon‐based nanozymes, including graphene, graphdiyne oxide, and carbon dots, offer tunability, scalability, and biocompatibility, and often serve as scaffolds for hybrid composites with metal oxides or noble metals [8, 9, 10, 11]. These examples demonstrate that nanozyme performance is more closely dictated by the rational control of composition, nanoarchitectonics, and hybridization to achieve specific catalytic outcomes than by a single material class.

Although nanozymes have advanced into multifunctional platforms with diagnostic and therapeutic potential [4, 12, 13, 14, 15], their utility remains limited by poor spatial targeting and by challenges operating in confined environments (such as root canal systems and synovial joint spaces), because the delivery of short‐lived reactive oxygen species (ROS) largely relies on passive diffusion [6, 16]. In complex biological settings, these shortcomings yield suboptimal reactivity, off‐target effects, and limited therapeutic efficiency [7, 17, 18]. Addressing these limitations requires integrating mobility, controllability, and environmental adaptability in addition to chemical activity.

Nanozyme microrobots have emerged as a new class of catalytic platforms to enable these properties. Microrobots are miniaturized systems, typically ranging from the micrometer to sub‐millimeter scale that can be actuated remotely by external stimuli, such as magnetic, acoustic, or optical fields, or intrinsically through internal properties triggered by chemical reactions [19, 20, 21]. The field has progressed from proof‐of‐concept systems to engineered micro‐ and nanoscale machines capable of navigating viscous fluids, tissues, and anatomically constrained spaces [19, 21, 22, 23, 24]. While microrobots offer precise navigation and control, they typically lack biochemical functionality. The convergence of nanozyme catalysis with programmable microrobotic mobility creates hybrid platforms for spatially and temporally resolved catalysis, enabling site‐specific and dynamically tunable interventions in precision medicine [25, 26, 27].

In this review, we highlight the expanding applications of nanozyme microrobots as they transition from proof‐of‐concept to practical biomedical solutions. We examine nanozyme catalysis from a biomechanical perspective, outlining how robotic actuation and control regulate kinetics, spatial targeting, and overall versatility. Therapeutically, localized generation of ROS enables targeted biofilm eradication, disruption of tumor microenvironments, and treatment of pneumonia, as well as other localized therapeutic interventions, with unprecedented spatial precision [25, 26, 28, 29, 30]. In diagnostics, catalytic reporters and imaging contrast agents support real‐time monitoring of biodistribution and treatment efficacy [28, 31]. We also discuss translational challenges, including scalable manufacturing, automated control, efficient in vivo real‐time tracking and delivery, and rigorous preclinical validation, and offer perspectives for clinical translation.

## 2 Foundations of Nanozyme Microrobots

### 2.1 Types of Catalytic Behaviors in Nanozymes

The catalytic repertoire of nanozymes emphasizes their growing significance as artificial enzyme mimics and lays the groundwork for their integration into more complex functional systems. Unlike natural enzymes, whose activity depends on delicate tertiary structures and narrow operational windows, nanozymes exhibit their catalytic behaviors from their intrinsic composition, crystal structure, and surface chemistry [4, 5, 7, 32]. Although nanozymes generally lack the high substrate selectivity characteristic of natural enzymes, they are more robust and tunable under diverse conditions [18, 33, 34]. As such, nanozymes offer a versatile platform for catalysis in environments where natural enzymes are unstable or inefficient.

The most widely exploited catalytic behaviors are peroxidase‐, oxidase‐, catalase‐, and superoxide dismutase‐mimicking activities, each associated with distinct material classes and reaction pathways [33]. Peroxidase‐like activity is generally observed in transition metal oxides such as Fe_3_O_4_ and Co_3_O_4_, where redox‐active metal centers catalyze the decomposition of hydrogen peroxide (H_2_O_2_) into hydroxyl radicals (•OH) [4, 32]. The catalytic efficiency depends strongly on the oxidation state cycling (e.g., Fe^2^^+^/Fe^3^^+^), the density of surface defects, and the availability of surface hydroxyl groups that stabilize radical intermediates. These mechanistic features explain the widespread use of Fe_3_O_4_ nanoparticles in biomedical applications involving ROS generation [4, 13, 15]. Oxidase‐like activity, in contrast, relies on the direct activation of molecular oxygen to oxidize organic substrates. MnO_2_ nanostructures and noble metal nanoparticles (Au, Pt) excel in this function due to their ability to stabilize O_2_ adsorption and lower the activation barrier for electron transfer [35]. In tumor therapy, this function has been exploited to modulate hypoxic environments via O_2_ consumption, whereas in environmental catalysis, it enables oxidation of recalcitrant pollutants [36, 37, 38].

Catalase‐mimicking activity illustrates the dual role of nanozymes as both catalytic and functional materials. Materials such as MnO_2_ and Fe_3_O_4_ can decompose H_2_O_2_ into H_2_O and O_2_, simultaneously reducing oxidative stress and generating oxygen bubbles. The efficiency of this process is governed by the availability of lattice oxygen and the dynamic redox cycling of the metal centers. Catalase‐like activity is attractive not only for biochemical applications but also as a sustainable strategy for O_2_ generation and as a driving mechanism for fuel‐free catalytic systems. Superoxide dismutase‐like activity further broadens the spectrum. For example, CeO_2_, with its Ce^3^^+^/Ce^4^^+^ redox pair and abundant oxygen vacancies, is an effective mimic. The reversible binding and release of oxygen species enables the disproportionation of superoxide radicals, supporting redox homeostasis and modulation of inflammatory diseases [39].

### 2.2 Applications and Unmet Challenges in Nanozymes

Biomedical applications have been a major driver in the development of nanozymes [7]. Earlier studies showed that Fe_3_O_4_ nanoparticles exhibit peroxidase‐like activity, catalyzing H_2_O_2_ to ROS in acidic pH and disrupting *Streptococcus mutans*‐induced biofilms and dental caries development in vivo, while sparing surrounding tissues [40, 41]. Building on this concept, MnO_2_‐based nanozymes have gained prominence in oncology, where their catalase‐like activity decomposes H_2_O_2_ into O_2_, alleviating tumor hypoxia and enhancing photodynamic therapy [42, 43, 44]. MnO_2_‐loaded nanocarriers have improved intratumoral oxygenation and therapeutic efficacy in mouse xenograft models, and several Mn‐based nanozyme formulations are advancing toward preclinical evaluation [45, 46]. Similarly, CeO_2_ nanozymes, which exhibit both superoxide dismutase‐ and catalase‐like activities, show promise as neuroprotective and anti‐inflammatory therapeutics [47, 48].

Diagnostic applications leverage the stability and tunability of nanozymes as robust alternatives to natural enzymes [12, 49]. Au and Pt nanozymes with oxidase‐ and peroxidase‐like activities have been integrated into colorimetric assays for glucose detection, maintaining activity under conditions that rapidly denature horseradish peroxidase [50, 51, 52, 53]. Carbon nanozymes have also been incorporated into paper‐based, point‐of‐care sensors, enabling affordable detection of biomarkers such as uric acid and cholesterol in clinical samples [54, 55].

Despite these advances, most nanozymes are either immobilized or freely dispersed, relying on passive diffusion to deliver substrates. As a result, their reactivity is limited by a lack of spatial precision, reduced performance in confined/inacessible spaces, and limited controllability after deployment. Achieving precise control remains a major obstacle to widespread use of nanozyme catalysis. While nanozymes can be functionalized with targeting ligands or embedded in carriers to enhance localization, their catalytic activity often lacks control over space and time. Off‐target reactions, especially those resulting from indiscriminate ROS production, pose risks of collateral damage to healthy cells and tissues. Attaining precise, on‐demand activation within specific microenvironments remains a challenge.

Restricted substrate accessibility further limits efficacy. In dense biofilms or hypoxic solid tumors, passive diffusion of ROS to target sites is inefficient. While morphological tuning and surface engineering can enhance activity, nanozymes alone cannot overcome transport barriers, leading to underperformance precisely where intervention is most needed. Limited adaptability to dynamic environments poses an additional challenge. Unlike natural enzymes that regulate activity through conformational or allosteric mechanisms, most nanozymes exhibit fixed reactivity [34, 56, 57]. Developing nanozymes that reach inacessible sites and sense and adapt to fluctuating microenvironments, activating catalysis selectively under pathological conditions, remains a critical frontier for the field.

### 2.3 Progress in Robotics at Small Scales

Microrobotics has rapidly emerged as a dynamic field focused on engineering micro‐ and nanoscale systems capable of active navigation and task execution in complex environments [19, 21, 58]. At these length scales, the motion of microrobots is governed by physical principles distinct from those dominating macroscopic systems, where inertia prevails [59]. Instead, microrobots operate in low Reynolds number regimes dominated by viscous forces, requiring locomotion strategies that overcome substantial drag and Brownian fluctuations [21]. Two general modes of motion have emerged: self‐propulsion and external (field‐driven) actuation.

Self‐propelled microrobots harness chemical gradients in their environment to generate movement. Catalytic microrobots are a prominent example, relying on local reactions such as H_2_O_2_ decomposition by catalytic coatings to produce gas bubbles or chemical gradients that drive propulsion [60, 61]. Chemical gradients can induce motion through diffusiophoresis or chemotaxis‐inspired mechanisms. While conceptually simple, self‐propulsion often requires fuel incompatible with physiological conditions and typically affords limited navigational control.

By contrast, externally actuated microrobots rely on applied fields to achieve controlled motion. Magnetic fields are among the most versatile and biocompatible, enabling untethered, fuel‐free actuation with deep tissue penetration and precise control via magnetic forces and torques [21]. Acoustic actuation employs ultrasound waves to trap, propel, or rotate microrobots, providing efficient propulsion in biological fluids and facilitating collective manipulation at larger scales [62]. Optical actuation harnesses light‐responsive materials such as gold nanostructures or photocatalysts, which convert light energy into localized heating or charge separation for propulsion [63]. Electric fields enable highly precise motion control via electrophoresis or dielectrophoresis, particularly in microfluidic environments [64].

Overall, microrobots offer sophisticated mobility and precise external control through magnetic, acoustic, optical, and electric actuation. However, their capabilities are largely confined to mechanical manipulation or cargo transport, lacking the intrinsic biochemical activity required for direct therapeutic or diagnostic functions. As a result, they often operate primarily as transporters or mechanical actuators, rather than as active agents capable of performing chemical transformations.

### 2.4 Convergence of Nanozymes and Robotics

**FIGURE 1:**
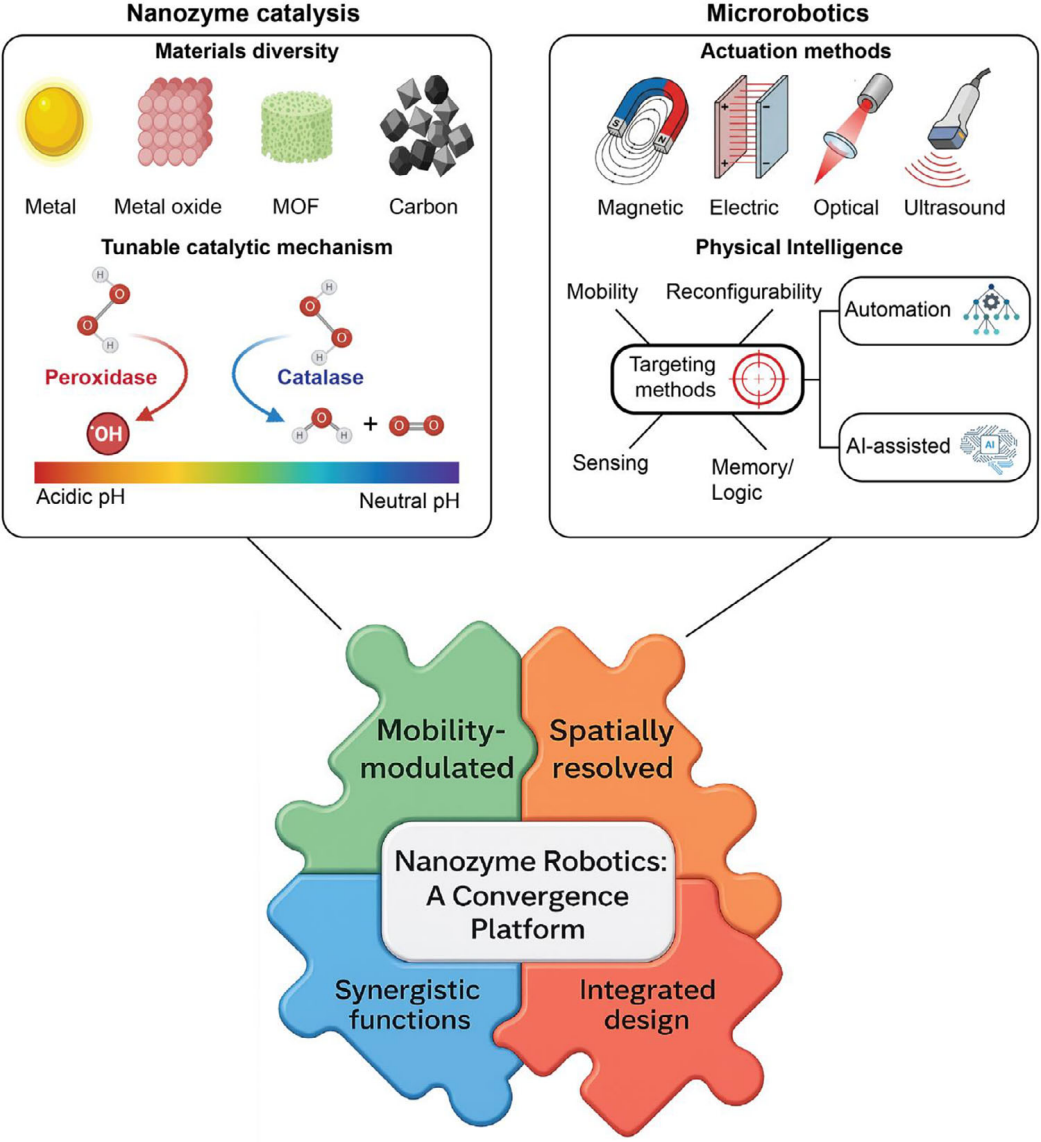
Microrobotics as a convergence platform for nanozyme functions. (Left) Nanozyme catalysis can be achieved with diverse materials, including metals, metal oxides, metal–organic frameworks (MOFs), and carbon‐based nanomaterials, together with their tunable catalytic mechanisms. (Right) Microrobotics provide multiple actuation methods (magnetic, electric, optical, ultrasound) and the concept of physical intelligence, encompassing mobility, reconfigurability, sensing, memory/logic, and targeting strategies. (Bottom) The convergence of these two domains yields nanozyme microrobots, where catalytic function is dynamically regulated by robotic motion and control. This integration enables mobility‐modulated and spatially resolved catalysis, supports integrated system design, and synergistic multifunctionality that facilitates translatable systems for practical applications, forming a unified platform for intelligent, controllable nanozyme‐based technologies.

Nanozymes and microrobots have each become well‐established, robust research domains, yet both face intrinsic limitations that restrict their individual impact. By leveraging design principles based on surface chemistry, nanoscale structure, and hierarchical assembly, nanozyme microrobots fuse catalytic and robotic functions into a cohesive platform where reactivity and motion are mutually reinforcing (Figure 1) [25]. Although nanozymes span a broad range of material classes, including carbon‐based and metal oxide framework‐derived systems, the present review focuses on metal oxide‐based nanozymes because current microrobotic actuation and control strategies rely heavily on magnetically, optically, or acoustically responsive materials; extending nanozyme microrobots to other catalytic classes through hybrid, doped, or composite architectures represents an exciting direction for future research.

Nanozymes can be integrated into microrobotic structures during their fabrication via self‐assembly, microfluidic synthesis, and additive manufacturing. Advances in fabrication techniques have enabled increasingly complex architectures. Microfluidic templating provides precise control over size, shape, and surface functionality, enabling the generation of uniform microrobots with tailored compartments and material distributions. For example, double emulsion‐microfluidic templating has produced hollow microrobots with a nanozyme shell for sustained ROS generation in confined spaces [26]. Additive manufacturing and lithographic methods further expand design freedom, facilitating the creation of complex architectures such as helical swimmers, hollow carriers, and soft, reconfigurable bodies [65]. These strategies not only support structural and functional diversity but also enable the seamless integration of auxiliary components, including magnetic nanoparticles, optical absorbers, and catalytic agents, to achieve multifunctional nanozyme‐based robotic systems. Asymmetric surface coating techniques can also create Janus microrobots in which one face is catalytically active. At the same time, the other remains inert, enabling directional propulsion under chemical gradients [66].

Through this convergence, catalysis evolves from a static, diffusion‐limited process into a spatiotemporally programmable system, empowering therapeutic and diagnostic interventions with precision and adaptability [67]. Navigation allows nanozymes to reach otherwise inaccessible environments such as deep biofilm layers, hypoxic tumor zones, and narrow porous media. Controlled motion improves substrate accessibility, enhances mixing at the reaction interface, and maintains a high local reactant concentration, thereby overcoming mass‐transport limitations that constrain traditional catalytic systems.

By examining how structural and chemical parameters govern both catalytic activity and robotic behavior, we identify design principles that enable the development of programmable, multifunctional nanozyme‐based robotic systems. Particular emphasis is placed on dynamic control strategies in which actuation and navigation modulate the spatial and temporal generation of ROS at target sites in real time [25, 26, 31]. Together, these features define nanozyme robotics as a unified convergence platform in which catalysis and mobility synergistically reinforce one another. The outcome is a new class of programmable, multifunctional systems that couple the chemical reactivity of catalytic nanomaterials with the adaptive control of robotic motion. This integrative approach broadens the functional landscape of both fields and opens opportunities for precision medicine, diagnostics, and responsive therapeutic interventions (Table 1).

**TABLE 1:** Summary of representative nanozyme robotic systems highlighting material design, actuation mode, disease model, and therapeutic mechanism.

System	Nanozyme composition & structure	Actuating force	Primary function	Disease setting	Key outcome	Reference
Catalytic antimicrobial robots	Fe_3_O_4_ nanoparticles assembled into aggregated structures or 3D molded helicoid	Magnetic field	Spatially resolved mechano‐catalysis	Biofilms in vitro and *ex* vivo	Mechanical biofilm removal and localized ROS generation via peroxidase‐activity for antibiofilm action	[67]
Nanozyme robotic superstructure	Fe_3_O_4_ nanoparticles assembled into magnetic bristle‐like superstructures	Magnetic field	Spatially resolved mechano‐catalysis	Dental biofilm (in vitro*, ex* vivo)	‐ Magnetic reconfiguration enables targeted mechanical removal and catalysis‐based killing ‐ Localized ROS generation via peroxidase‐like activity and active mixing	[31]
Nanozyme‐shelled microcapsule	Hollow microcapsules with Fe_3_O_4_ nanozyme shell	Magnetic field	Spatially resolved catalysis	Root‐canal biofilm (in vitro)	‐ Precise navigation through bifurcated canals ‐ Localized biofilm eradication via on‐site ROS generation	[26]
Dabbing nanozyme microrobot	Fe_3_O_4_ nanoparticles	Magnetic field	‐ Spatially resolved catalysis ‐ Mobility‐modulated catalysis	Fungal biofilm (in vitro*, ex* vivo)	‐ Programmable dabbing motion for localized nanoparticle deposition ‐ Complete fungal killing via localized ROS delivery	[25]
Photomagnetic Janus microrobot	Cu‐doped BiOI photocatalyst on magnetic Fe_3_O_4_ core	Combined visible light + magnetic guidance	Dual photo‐ and magneto‐catalytic therapy	Sinusitis model (*in* vivo)	Fiber‐assisted activation and magnetic navigation achieve biofilm clearance and tissue recovery	[68]
Helical micromachine	Graphene oxide‐templated Fe_2_O_3_ helix	Magnetic field	ROS‐based catalytic disinfection under endoscopic guidance	Human cadaver ear (T‐tube *ex* vivo)	‐ Mechanical‐chemical synergy removes MRSA biofilm ‐ Safe retrieval under endoscope visualization	[69]
Magnetic hydrogel micromachine	Fe_3_O_4_‐loaded PNIPAM hydrogel absorbing H_2_O_2_	Magnetic + thermal (hyperthermia)	On‐demand catalytic release	Biofilm (in vitro)	Magnetothermal heating triggers controlled H_2_O_2_ release and ROS activation for biofilm disruption	[70]
Ultrasound‐responsive catalytic microbubble	Fe_3_O_4_‐based piezoelectric gas‐core nanostructure	Ultrasound	Cavitation‐enhanced catalysis	Lung infection model (in vitro)	Dual chemical‐mechanical disruption of biofilm with enhanced ROS penetration	[71]
Heterojunction nanozyme	CuFe_2_O_4_‐MoS_2_ quantum dot nanoheterojunction	Ultrasound	Dynamic Fenton‐like catalysis and bone regeneration	Bone infection model (in vivo)	‐ Ultrasound‐induced Fe^3^^+^/Fe^2^^+^ cycling activates ROS ‐ Promotes osseointegration and antibacterial effect	[72]
Janus nanocatalytic robot	Pt catalytic cap on Fe_3_O_4_@SiO_2_ core	Chemical propulsion (H_2_O_2_) + magnetic guidance	Tumor catalytic therapy	Mouse tumor (in vitro)	‐ Self‐propulsion enhances ROS‐mediated tumor inhibition ‐ MRI‐guided navigation	[66]

## 3 Working Principles of Nanozyme Microrobots

In principle, nanozyme robotics combines catalytic activity with robotic control, enabling on‐demand and site‐specific reactions. To achieve such programmable mobility, a variety of energy inputs have been employed, including acoustic, electric, optical, and magnetic fields (Table 2) [21]. Among these, magnetic fields are particularly advantageous, offering untethered, fuel‐free operation with deep tissue penetration, minimal cellular damage, and precise tunability through diverse field configurations [20, 73]. Magnetic actuation also provides retrievability for safety, broad material compatibility, and straightforward integration with other propulsion mechanisms [19]. Beyond navigation, magnetic fields can generate mechanical forces, trigger the release of drugs, and induce localized heating (hyperthermia) for therapeutic or diagnostic purposes [19]. Owing to these capabilities, magnetic microrobots have emerged as one of the most versatile and clinically promising strategies for precision biomedical applications [24].

**TABLE 2:** Comparison of actuation and control modalities for nanozyme microrobotics using standardized physical parameters and benchmarkable performance metrics.

Modality	Control inputs	Penetration in tissue	Spatial control metrics	Temporal control metrics	Coupling to nanozyme function	Safety / side effects	Benchmark items
**Magnetic field**	Field amplitude **B** (mT), gradient **∇B** (T m^−^^1^), frequency **f** (Hz), waveform (RMF/gradient/pulsed)	cm‐scale	Path tracking error (µm–mm), targeting radius (mm), steering DOF (2D/3D), retrieval success (%)	Response time to reorientation (s), closed‐loop update rate (Hz)	Navigation, reconfiguration, contact mechanics, mixing enhancement; can co‐enable magnetic hyperthermia when applicable	Temperature rise **ΔT** (°C), when heating occurs; off‐target displacement (mm)	(i) B, ∇B, f; (ii) depth tested; (iii) targeting accuracy; (iv) performance ratio actuated vs static (e.g., ROS, biofilm removal)
**Ultrasound**	Frequency (MHz), acoustic pressure (MPa) or intensity (W cm^−^^2^), duty cycle (%), exposure time (s)	cm‐scale	Focal spot size (mm), actuation volume (mm^3^), localization vs bulk activation	On/off kinetics (s), pulse response, cavitation threshold crossing	Cavitation‐assisted mixing, permeability enhancement, mechanochemical synergy; can trigger piezo/sono‐catalysis in specific materials	Temperature rise ΔT (°C), evidence of cavitation damage	(i) MHz, W cm^−^^2^ (or MPa), duty; (ii) focal size; (iii) ΔT/MI; (iv) quantitative enhancement vs control; (v) tissue damage assessment endpoint
**Light**	Wavelength (nm), irradiance (mW cm^−^^2^), dose (J cm^−^^2^), spot size (mm)	mm‐scale	Optical spot size (mm), effective activation depth (mm), confinement index (activated volume/total)	Switching speed (ms–s), photobleaching time (min), dose‐dependent kinetics	On‐demand photocatalysis, local ROS generation, photothermal assist	Phototoxicity endpoints (cell viability %, inflammatory markers), ΔT (°C) for photothermal components	(i) nm, mW cm^−^^2^, J cm^−^^2^; (ii) depth; (iii) activation volume; (iv) phototoxicity metric; (v) actuated vs non‐actuated catalytic output
**Chemical propulsion**	Fuel identity + concentration (e.g., H_2_O_2_ %, mM), ionic strength, pH; catalyst loading (mg mL^−^^1^ or wt.%)	Local only (direct access; report diffusion distance/time if relevant)	Directionality metric (chemotaxis index), velocity (µm s^−^^1^), trajectory persistence	Start/stop time (s), depletion time (min), performance decay half‐life	High‐speed motion; mixing/transport enhancement via self‐propulsion; can amplify mass transport to catalytic sites	Fuel toxicity (viability %, hemolysis), byproduct concentration, local pH shift	(i) Fuel type/concentration; (ii) speed; (iii) lifetime; (iv) toxicity endpoint; (v) catalytic enhancement vs static under matched fuel

### 3.1 Mobility‐Modulated Catalysis

**FIGURE 2:**
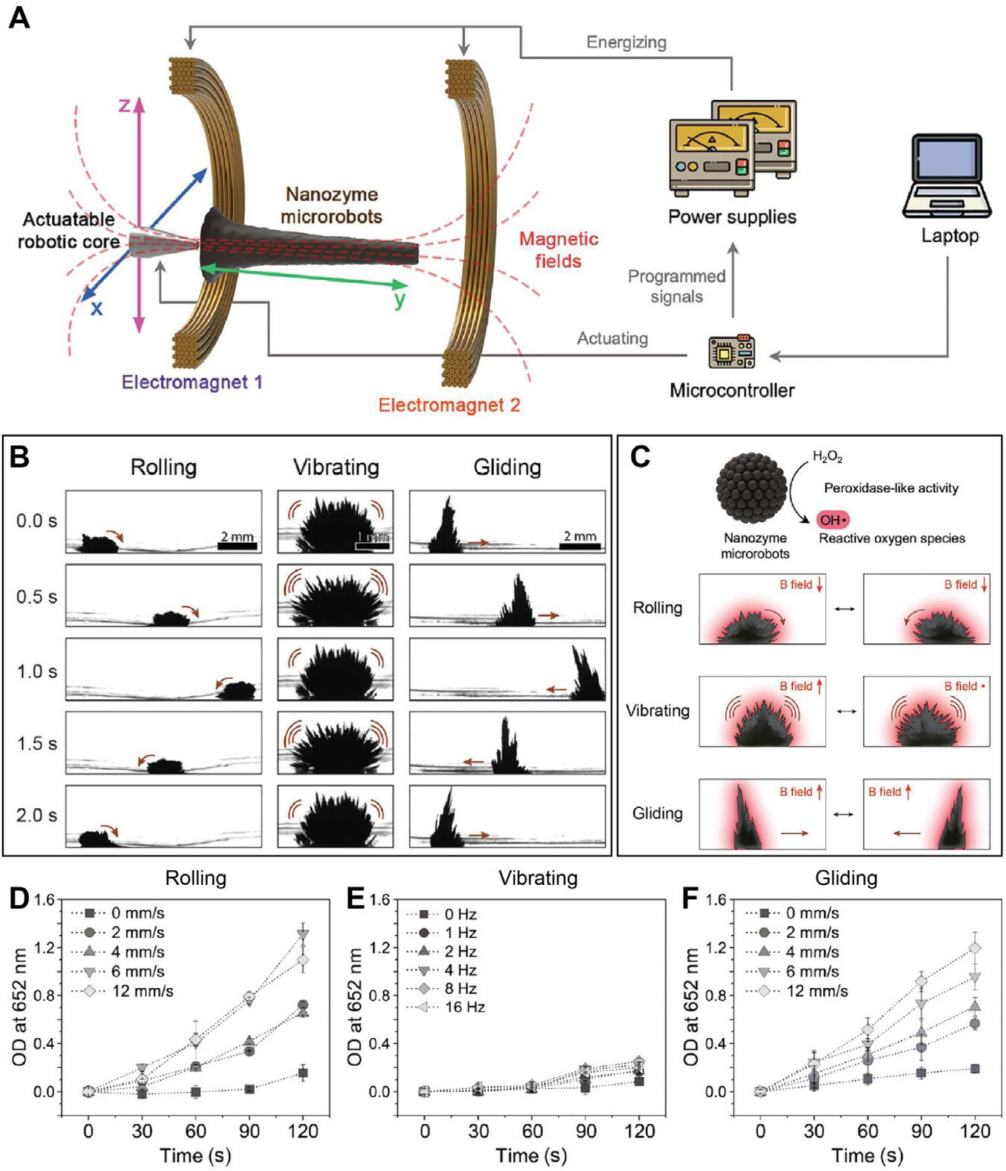
Mobility‐modulated catalysis. (A) Assembly, control, and functional properties of robotic nanozyme assemblies. (B) Their mode of motion. (C) Catalytic activity in situ generated by motion dynamics. The 3,3′,5,5′‐tetramethylbenzidine (TMB) assay demonstrates the on‐site generation of ROS from H_2_O_2_ by the catalytically active (peroxidase‐like) nanozyme microrobots. Catalytic activity dynamics of (D) rolling, (E) vibrating, and (F) gliding motions. Adapted from Ref [25] under the Creative Commons CC‐BY‐NC‐ND license.

Mobility‐modulated catalysis represents a key advantage of nanozyme robotic systems, enabling catalytic activity to be influenced not only by material composition and structure but also by controlled motion [74]. At micro‐ and nanoscales, mass transport is dominated by diffusion; thus, catalytic reactions often operate in a diffusion‐limited regime where substrate replenishment is slow [75]. Robotic mobility can shift these systems toward convection‐enhanced transport by inducing convective mixing in the fluid boundary around the microrobot, which stirs and increases substrate flux toward active sites. The effect can be qualitatively described by transport parameters such as the Péclet number (Pe), which reflects the balance between advective transport and diffusion. When microrobots generate local flow fields through their motion, they effectively increase Pe, thereby increasing reaction rates through more frequent interactions between catalytic surfaces and H_2_O_2_, accelerating ROS generation. For example, magnetically actuated Fe_3_O_4_‐based microrobots not only act as static catalysts but as motion‐activated reactors whose chemical output is tuned by the locomotion mode, velocity, and trajectory [25]. Programming the magnetic field to generate distinct locomotion modes, such as rolling, gliding, vibrating, and dabbing, demonstrates a direct coupling between microrobot mechanics and catalytic performance (Figure 2A–C). Rolling and gliding generate large‐scale convective mixing, which enhances substrate transport and ROS dispersal, thereby increasing reaction rates several‐fold compared to static systems (Figure 2D,F). In contrast, localized vibration and dabbing produce confined mixing and correspondingly localized catalytic effects (Figure 2E). This dynamic interplay highlights the synergy between mechanical agitation and chemical reactivity, transforming catalysis from a static process into an active, adaptive, and precisely controllable function.

### 3.2 Spatially Resolved Catalysis

Spatially resolved catalysis in nanozyme microrobots refers to the ability to control where catalytic reactions occur. Unlike conventional nanozyme systems, which rely on passive diffusion and therefore distribute catalytic activity broadly and unpredictably, nanozyme microrobots introduce spatial precision through an interplay of navigation, activation, and environment‐dependent reactivity [61]. Conceptually, spatially resolved catalysis can be defined by two interdependent elements: (i) Active positioning of catalytic materials and (ii) Spatial confinement of catalytic flux.

Catalysis‐driven propulsion is among the earliest and most widely studied mechanisms for microrobotic propulsion [21, 76]. Catalytic materials such as Pt, MnO_2_, and related coatings decompose H_2_O_2_ to generate oxygen bubbles that propel microrobots. This bubble propulsion has been used for targeted cargo transport and pollutant degradation. Beyond bubble‐based motion, catalytic reactions can establish self‐generated chemical gradients that bias particle movement via diffusiophoresis. For example, microrobots with asymmetric catalytic coatings form local product gradients, enabling directional movement without the need for external fields [77, 78]. These systems demonstrate how catalytic reactions can serve as local energy sources for autonomous mobility, with motion in turn optimizing the spatial distribution and utilization of catalytic sites. Thus, mobility enhances catalysis by actively overcoming the diffusion limitations that restrict nanozyme performance, especially slow substrate transport and poor access in confined or heterogeneous environments.

**FIGURE 3:**
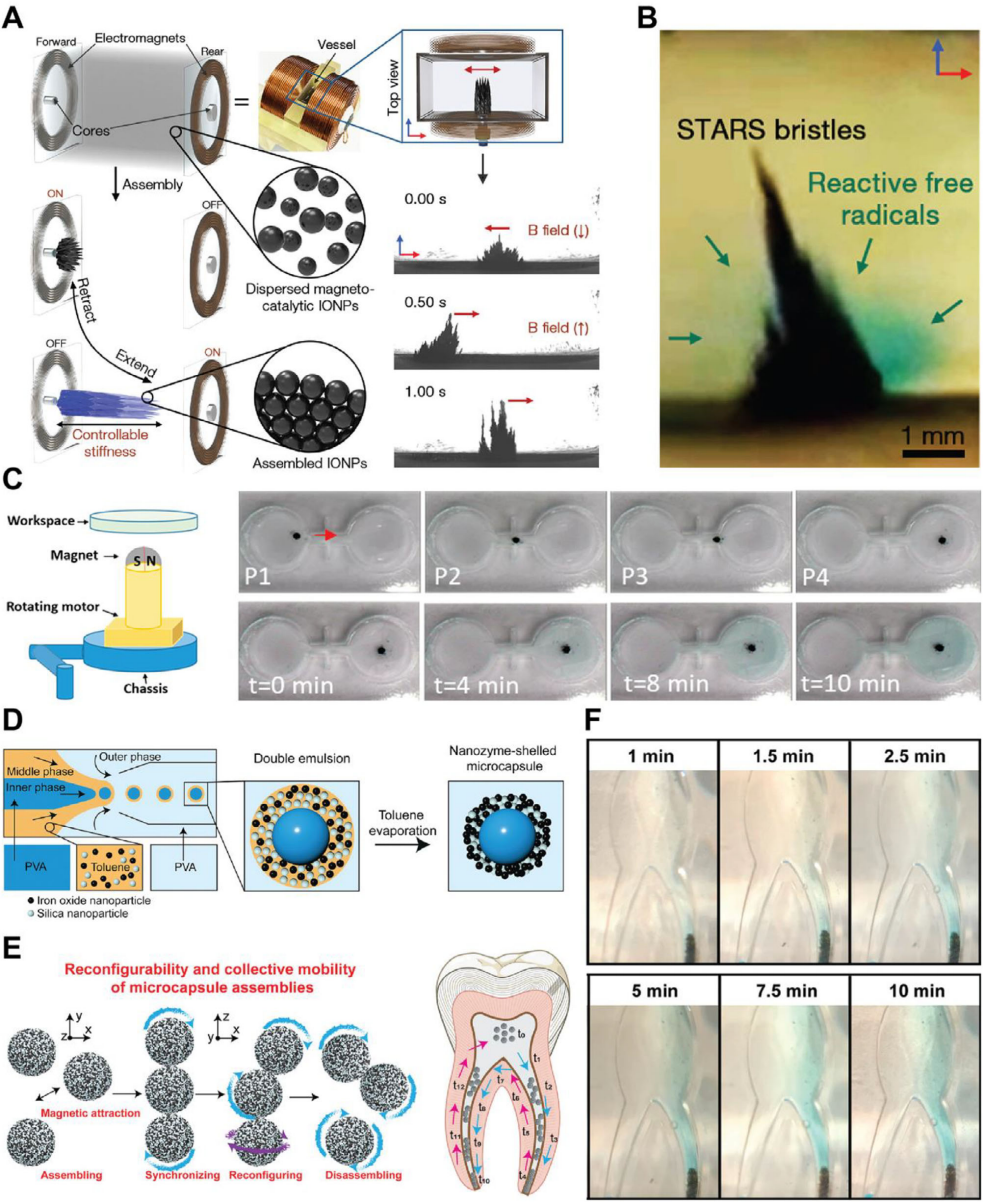
Spatially resolved catalysis. (A) Assembly, magnetic control, and functional properties of Fe_3_O_4_ nanoparticles‐based robotic superstructures. (B) Catalytic activity showing localized ROS generation around the superstructure, visualized by a 3,3′,5,5′‐tetramethylbenzidine (TMB) colorimetric assay. Adapted with Ref [31] under CC‐BY‐NC‐ND 4.0. (C) Schematic of the permanent magnet system used to generate a rotating magnetic field for targeted catalysis by Fe_3_O_4_ nanoparticle collectives. Sequential images (P1–P4) illustrate the movement of the nanoparticle collective over time and the progression of TMB catalysis from 0 to 10 min. Adapted with permission from Ref [80] (Copyright 2021, American Chemical Society). (D) Fabrication of nanozyme‐shelled microcapsules via a droplet‐templated microfluidic method. (E) Collective navigation of nanozyme‐shelled microcapsule assemblies within a bifurcated root canal model. (F) Targeted catalysis of nanozyme‐shelled microcapsules, showing localized catalytic activity restricted to one canal, as determined by the TMB assay. Adapted from Ref [26] under the Creative Commons CC‐BY‐NC‐ND license.

For precision medicine, it is essential to confine reactivity to specific sites, allowing potent biochemical effects to be localized for maximum efficacy and minimal collateral impact. By merging catalysis with robotic control, nanozyme robotics enables mobile, programmable systems that direct and modulate reactivity with high spatial and temporal precision. In this context, magnetic fields provide precise, non‐invasive control over navigation, allowing microrobots to reach deep tissue sites such as root canals or vascular networks [27]. In addition, magnetic‐field‐driven self‐assembly organizes nanoparticles into dynamic, reconfigurable microrobotic swarms whose collective behavior enhances locomotion, adaptability, and catalytic performance [79]. For instance, Fe_3_O_4_ nanoparticles assemble into bristle‐like superstructures that continuously adapt to applied magnetic fields; beyond mechanical motion, this control precisely positions catalytic sites, enabling selective ROS generation around the superstructures in the presence of H_2_O_2_ (Figure 3A,B) [31]. Similarly, assemblies of Fe_3_O_4_ nanoparticles guided by a rotating magnetic field can be transported to designated locations to perform localized catalysis (Figure 3C) [80], achieving on‐site production of •OH radicals.

Recent efforts to achieve spatial control in anatomically relevant settings have produced nanozyme microrobots that operate within complex biomedical topographies. In one system, Fe_3_O_4_ nanoparticles were confined within the shell of microcapsules fabricated by double‐emulsion microfluidics (Figure 3D). Under external magnetic field actuation, these microcapsule‐based nanozyme microrobots exhibit collective, programmable motion and navigate branched or confined regions of root canals (Figure 3E) [26]. They display self‐adaptive behavior, adopting zig‐zag configurations and reversible assembly‐disassembly modes, to traverse constricted geometries without clogging or loss of activity. Once guided to apical regions, the microrobots executed in situ catalysis (Figure 3F). This motion‐enabled catalytic control transforms catalysis from a passive diffusion‐driven process into an actively directed one, where reactivity is localized to predetermined sites.

Collectively, these studies demonstrate that, whether autonomous or field‐driven, nanozyme microrobots can overcome microenvironmental barriers through active navigation and localized catalysis. By combining catalytic activity with controllable mobility, they offer chemical precision and environmental adaptability, enabling reactions to occur exactly where and when needed. Spatially resolved catalysis thus provides a blueprint for precision therapies in which motion and reactivity are orchestrated within a unified, programmable framework.

### 3.3 Integrated Design of Nanozyme Robotics

The integrated design of nanozyme robotics provides a distinct advantage over conventional catalytic systems, which are often static, diffusion‐limited, and constrained by environmental conditions. Unlike traditional nanozymes that depend on the surrounding microenvironment for reactants and activation, these robotic systems combine catalytic, mechanical, and responsive components within a unified, programmable framework. Such integration allows nanozyme robots to perform precision catalysis while dynamically regulating chemical reactivity in response to physiological cues or external stimuli. This convergence of motion, navigation, and catalysis transforms nanozyme robotics into a dynamic and controllable platform that operates efficiently within complex biomedical environments.

**FIGURE 4:**
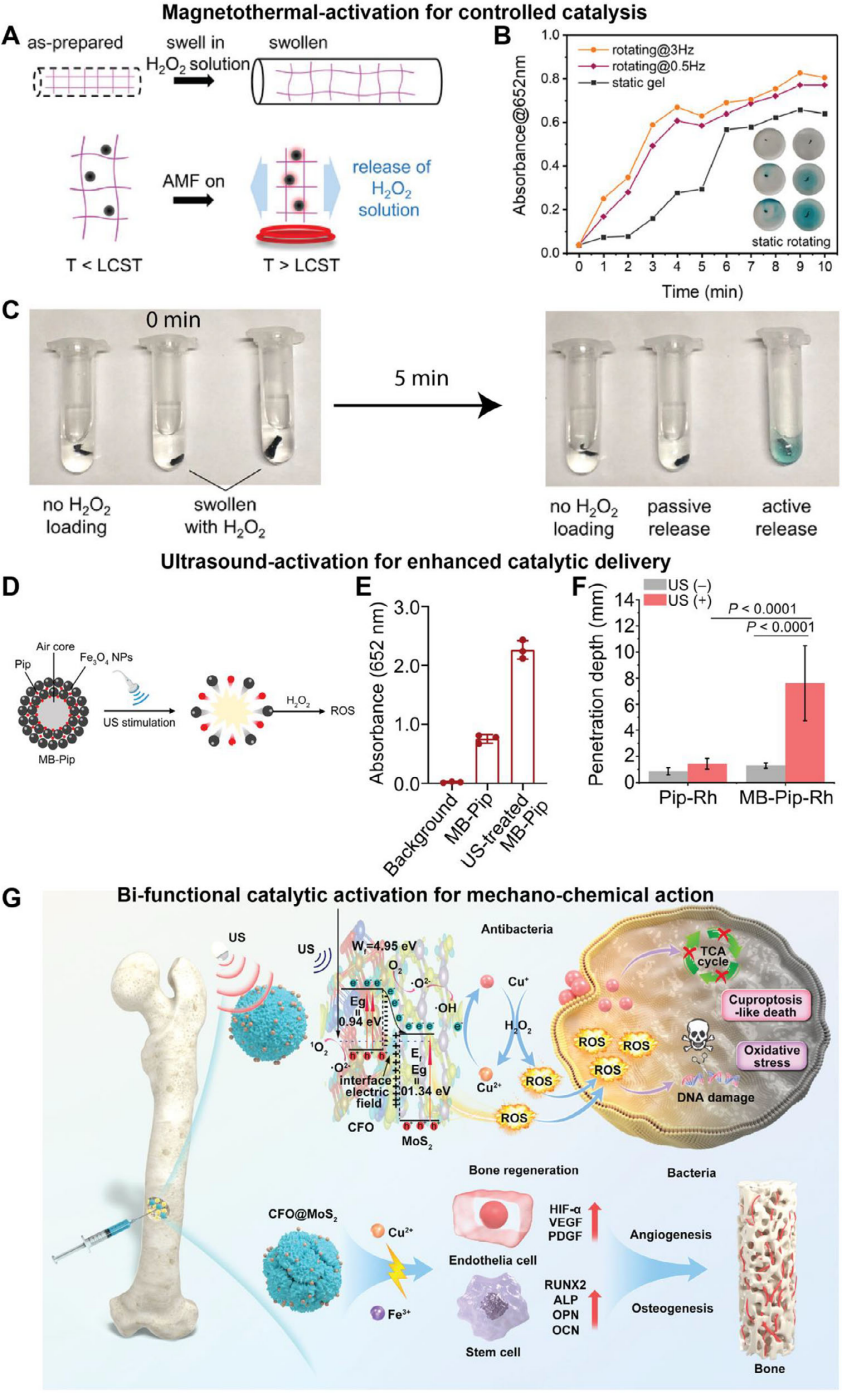
Integrated design of nanozyme robotics. (A) Formation of magnetic hydrogel micromachines (MHMs) capable of absorbing H_2_O_2_ solution and actively releasing the cargo when heated above the lower critical solution temperature (LCST). (B) Absorbance change of the TMB/H_2_O_2_ solution over time. Inset: snapshots of two experimental groups at 0, 3, and 5 min. (C) Comparison of active and passive release. The active group is heated via magnetothermal effect and actuated under a rotating magnetic field; the passive group reacts without heating or magnetic stimulation; the control group contains MHMs without H_2_O_2_ loading. Adapted from Ref [70] under the Creative Commons Attribution License. (D) Schematic illustration of ultrasound‐responsive catalytic microbubbles. (E) Peroxidase‐like catalytic activity of microbubbles before and after ultrasound treatment using the TMB assay. Pip‐Rh stands for piperacillin labeled with Rh. (F) Penetration depth of different samples within EPS‐mimicking gels. Adapted from Ref [71] under a Creative Commons Attribution NonCommercial License 4.0 (CC BY‐NC). (G) Schematic illustration of the ultrasound‐activated antibacterial mechanism and bone regeneration capability of the copper ferrite@MoS_2_ heterojunction. Redox‐driven Fenton catalysis accelerates ROS generation, while microstreaming enhances transport for synergistic mechanical–chemical action; Cu^2+^ released from CFO@MoS_2_ further promotes angiogenesis and osteogenesis. Adapted with permission from Ref [72] (Copyright 2025, Elsevier).

One key advantage of the integrated architecture is the ability to encapsulate and internally regulate reactants, such as H_2_O_2_, within the microrobot [70]. By confining H_2_O_2_ to an internal compartment, the microrobot generates ROS on‐site, bypassing the need for exogenous H_2_O_2_ (Figure 4A). Modularity also enables control of reaction kinetics through robotic mobility (Figure 4B). In addition, localized heating induced by the magnetic‐hyperthermia effect of Fe_3_O_4_ can serve as an on‐demand trigger to activate catalytic reactions (Figure 4C). Together, these mechanisms help maintain consistent catalytic output even in hypoxic or substrate‐depleted microenvironments that often limit the efficacy of traditional Fenton‐based nanozymes. The controlled internal release of H_2_O_2_ not only enhances temporal precision but also reduces collateral exposure by confining the generation of ROS to target sites.

The integrated framework further supports synergistic coupling with external energy sources, such as ultrasound, to trigger and regulate catalytic activity. For example, a modular gas‐core nanozyme robotic system has been integrated with ultrasound to enable multimodal catalytic control and enhanced therapeutic efficacy (Figure 4D) [71]. The platform combines Fe_3_O_4_ nanoparticles and piezoelectric components to link catalytic and mechanical responses under ultrasound stimulation (Figure 4E). It generates localized cavitation and microbubble oscillations that physically disrupt barriers such as dense biofilms, thereby improving ROS penetration and substrate accessibility (Figure 4F). This dual mechanism of chemical activation and mechanical disruption demonstrates how ultrasound functions as a control module in nanozyme robotic systems, enabling precise spatiotemporal regulation of catalytic activity and supporting the development of intelligent catalytic microrobots capable of on‐demand deep‐tissue therapeutic delivery.

In another example, under ultrasonic stimulation, certain transition‐metal nanozymes undergo valence‐state modulation, transiently shifting between oxidation states that activate Fenton‐like reactions (Figure 4G) [72]. Specifically, ultrasound serves as both an activation and modulation cue by inducing valence‐state transitions within the nanoheterojunction nanozyme composed of copper ferrite (CuFe_2_O_4_) and molybdenum disulfide (MoS_2_) quantum dots (CFO@MoS_2_), dynamically switching the Fe^3^^+^/Fe^2^^+^ redox cycle to trigger Fenton‐like reactions and accelerate ROS generation. The resulting microstreaming not only enhances ROS penetration but also improves reactant diffusion and substrate turnover, producing a dual mechanical‐chemical mode of action. In addition, Cu^2+^ released from CFO@MoS_2_ enhances the angiogenesis and osteogenesis capabilities.

Collectively, these capabilities demonstrate how nanozyme robotics integrates catalysis and actuation by combining internal fuel reservoirs, energy responsiveness, and environmental adaptability within a unified platform. The architecture affords design flexibility, allowing elements such as fuel encapsulation, ultrasound sensitivity, and catalytic tuning to be independently optimized for specific biomedical contexts. This integrative approach establishes a programmable, multifunctional platform that surpasses conventional nanozymes in spatial precision, reactivity control, and therapeutic performance.

## 4 Biomedical Applications of Nanozyme Robotics

The convergence of catalysis and robotics is being applied to biomedical interventions by turning passive, diffusion‐limited reactions into dynamic, controllable processes. This was exemplified by the seminal work that describes the use of catalytic Fe_3_O_4_ and magnetic actuation, termed CARs (Catalytic Antimicrobial Robots), for biofilm eradication [67]. The study shows how catalytic activity and robotics control can exert physico‐chemical biofilm killing and complete removal with microscale precision. Another example is a tubular TiO_2_ microrobot coated with Pt nanoparticles, which serve as both catalytic sites and bubble generators for the removal of dental biofilm [81]. Their motion improves substrate flux to catalytic sites, enhances mixing at the reaction interface, and maintains high local concentrations of reactive intermediates. By combining mechanical disruption with localized catalysis, these robots achieve rapid bubble‐driven propulsion and kill up to 95% of bacteria within 5 min. Their autonomous motion promotes fluid mixing and localized ROS generation, yielding a synergistic dual mechanism for biofilm disruption [81].

**FIGURE 5:**
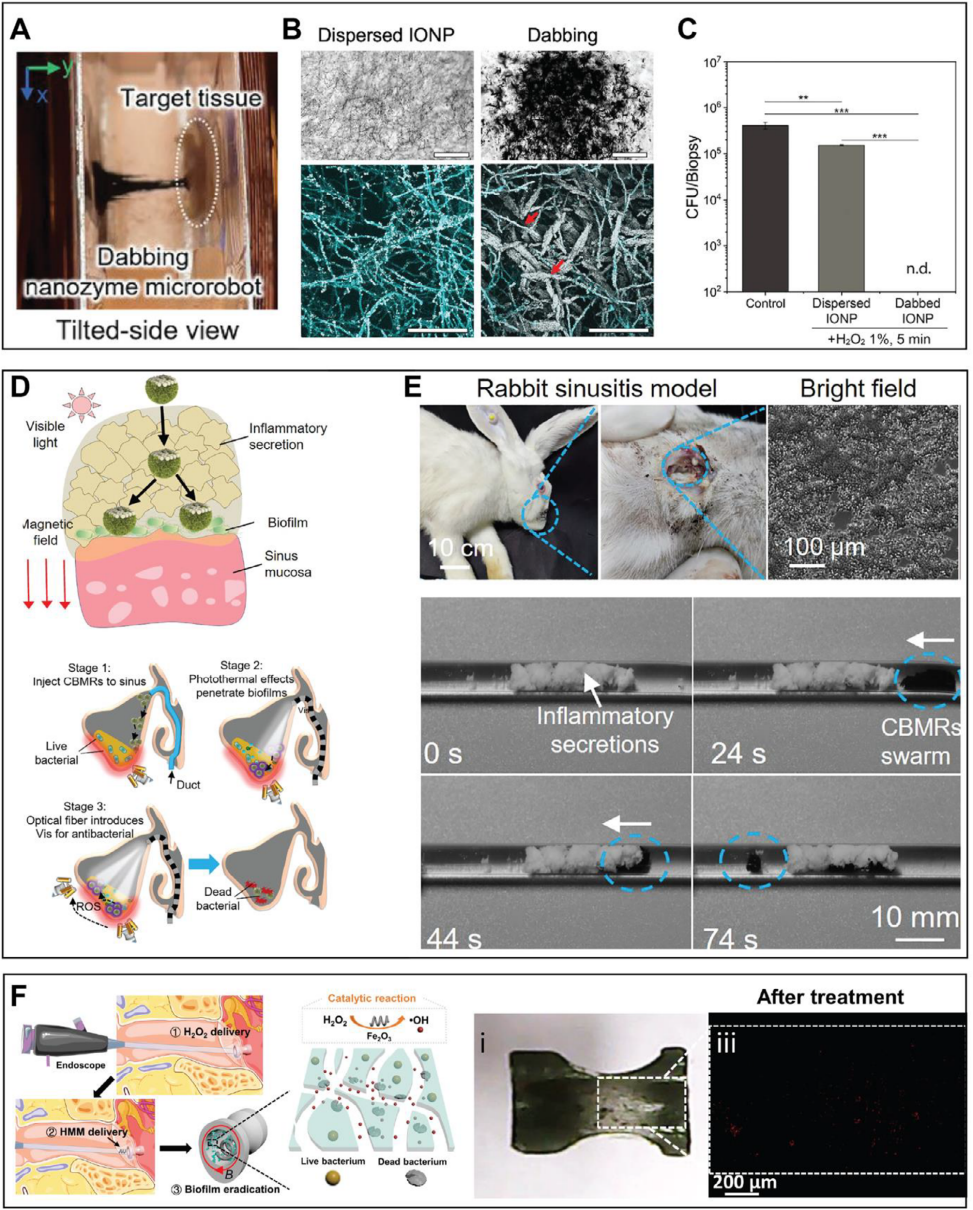
Biomedical applications of nanozyme robotics. (A) Precision programming and automation of the dabbing nanozyme microrobot, showing the superstructure extending and tapping the targeted surface. (B) Comparison of coating efficiency between dispersed Fe_3_O_4_ nanoparticles (IONP) treatment and IONP dabbing. Bright‐field microscopy (upper, scale bar: 500 µm) and confocal microscopy (lower, scale bar: 100 µm) reveal markedly higher IONP coating density on *C. albicans* biofilms following dabbing treatment. (C) Cell viability analysis showing limited killing of *C. albicans* cells by dispersed IONP treatment. Adapted from Ref [25] under the Creative Commons CC‐BY‐NC‐ND license. (D) Photomagnetic synergy enhances microrobot penetration through biological barriers. (E) Schematic and optical visualization of a rabbit sinusitis model demonstrating nanozyme robotic swarm penetration through biofilm and inflammatory secretions to reach the underlying sinus mucosa. Bright‐field and fluorescence imaging (red and green channels representing dead and live cells, respectively) illustrate inflammatory composition and time‐lapse tracking of swarm penetration under magnetic actuation. Adapted with permission from Ref [68] (Copyright 2025, The American Association for the Advancement of Science). (F) Schematic of the endoscope‐assisted biofilm eradication procedure using actuated Fe_2_O_3_ HMMs for tympanostomy tube disinfection. Top and cross‐sectional views of the T‐tube show biofilm morphology before and after treatment, with fluorescent imaging confirming efficient biofilm removal. Adapted from Ref [69] under a Creative Commons Attribution NonCommercial License 4.0 (CC BY‐NC).

Magnetically actuated Fe_3_O_4_ nanoparticles‐based microrobots not only generate ROS but also enable targeted catalytic treatment by directing ROS generation precisely at the infection site. Compared to static or freely dispersed nanoparticles, this active transport enhances degradation of the biofilm matrix. One example is a nanozyme robotic system programmed to exhibit dabbing motion for precision fungal treatment [25]. Using an electromagnet‐based setup, microrobots are guided to approach and contact target sites via stepwise linear extension modulated by a 15 Hz sine wave superimposed on a bias signal. This produces smooth, repeatable dabbing motions that precisely deposit dense layers of Fe_3_O_4_ nanozymes onto *Candida albicans* biofilms (Figure 5A), in contrast to the sparse binding observed with freely dispersed nanoparticles (Figure 5B). As a result, the dabbing microrobots achieve total fungal eradication, with no detectable viable cells, outperforming static treatments (Figure 5C).

Another example leverages the integrated design of nanozyme robotics to construct a photomagnetic microrobotic system, integrating Cu single‐atom‐doped BiOI (BiOI:Cu) photocatalysts with magnetic Fe_3_O_4_ cores in a Janus configuration [68]. This architecture enables magnetic navigation and light‐activated catalysis. Under visible‐light illumination, the Cu‐BiOI shell generates ROS and local photothermal heating, reducing mucus viscosity and enhancing microrobot mobility (Figure 5D). Guided by a fiber‐assisted magnetic control system, the microrobots achieve synchronized photomagnetic motion, penetrating viscous inflammatory barriers and disrupting biofilms through a combination of mechanical and catalytic actions.

The system's therapeutic performance was validated in an in vivo rabbit model of chronic maxillary sinusitis with biofilm infection. After intranasal injection, the microrobots were magnetically guided through pus‐like secretions, while fiber‐delivered light‐activated ROS generation at the infection site. X‐ray and CT imaging confirmed precise navigation and barrier penetration, and post‐treatment analyses showed complete biofilm clearance, epithelial recovery, and normalized inflammation, with no detectable tissue damage (Figure 5E). This photomagnetic synergy demonstrates the potential of dual actuation‐catalysis design for non‐invasive infection treatment in anatomically constrained spaces.

In another application, a magnetically actuated Fe_2_O_3_ helical micromachine (HMM) was developed for endoscope‐assisted removal of biofilm occlusions in tympanostomy tubes, marking a clinically relevant advance for nanozyme robotics in confined biological environments [69]. The Fe_2_O_3_ HMMs were fabricated via a microfluidic graphene oxide templating process to create helical structures with tunable geometry and intrinsic peroxidase‐like activity. Under rotating magnetic fields, HMMs exhibited three distinct motion modes (straight, tilted, and wobbling), with the wobbling mode generating the strongest hydrodynamic shear and convective mixing. This enhanced mechanical disruption and ROS diffusion enabled the rapid removal of methicillin‐resistant *S. aureus* biofilms at low H_2_O_2_ concentrations through a combination of physical agitation and catalytic action (Figure 5F). The system was validated in an ex vivo human cadaver ear model, where HMMs were magnetically guided under endoscopic visualization into a biofilm‐occluded T‐tube implanted in the tympanic membrane. The microrobots navigated through the narrow lumen, disrupted debris, and catalytically killed residual bacteria without damaging adjacent tissue. The HMMs were magnetically retrieved post‐treatment, demonstrating safety and controllable operation. This integrated platform, which combines magnetic actuation, catalytic disinfection, and endoscopic delivery, represents a promising approach to minimally invasive robotic disinfection in complex anatomical sites.

The programmability of nanozyme microrobots also extends to oncology, where precise navigation and localized catalysis can help overcome the hypoxic and acidic conditions that reduce the efficacy of conventional therapies. Magnetic nanozyme microrobots with catalase‐ or peroxidase‐like activities can decompose H_2_O_2_ into oxygen (via catalase‐like pathways) and ROS (via peroxidase‐like pathways), relieving hypoxia and enhancing oxidative stress within the tumor. Studies have shown that Fe_3_O_4_ nanoparticle‐based microrobots, guided by rotating magnetic fields, can penetrate tumor spheroids and locally amplify ROS production, thereby achieving synergistic effects with chemotherapy or photodynamic therapy [82, 83]. Magnetothermal actuation has also been used to couple catalytic ROS generation with localized heating, achieving dual‐mode tumor disruption while minimizing systemic toxicity [84]. For example, self‐propelled Janus nanocatalytic robots (JNRs), built from Pt‐coated Fe_3_O_4_@SiO_2_ nanoparticles, combine H_2_O_2_‐driven propulsion with localized ROS generation for catalytic therapy and real‐time magnetic resonance imaging (MRI) guidance [66]. In vivo results showed significant tumor inhibition, where mice treated with JNRs exhibited markedly reduced tumor volumes compared to controls, highlighting the synergistic benefit of propulsion‐enhanced delivery and catalytic performance.

Collectively, these studies highlight the emerging potential of nanozyme robotics as a multimodal, responsive, and clinically translatable platform for site‐specific, programmable biochemical interventions in both ex vivo and in vivo settings. These systems demonstrate precise spatial reactivity, deep‐tissue adaptability, and hydrodynamic mixing coupled with mechanical penetration, enabling localized catalysis under imaging or endoscopic control. The dynamic interplay between motion and catalysis defines the synergistic nature of nanozyme microrobots, in which mechanical actuation and chemical reactivity cooperate to enhance therapeutic efficacy.

## 5 Conclusion and Outlook

By integrating catalytic activity with mobility, programmability, and external control, nanozyme microrobots shift catalysis from a passive surface process to a dynamic, spatiotemporally regulated function. These platforms couple localized chemical reactivity with controlled motion and environmental responsiveness within a single programmable system, enabling on‐demand catalytic action with high spatial and temporal precision.

Future research should establish a quantitative framework to describe and predict spatiotemporal catalytic behavior. Integrating reaction‐diffusion theory with finite‐element simulations and machine‐learning optimization could enable predictive tuning of structural and dynamic parameters to maximize performance. Such approaches would reduce empirical trial‐and‐error and uncover design principles linking catalytic kinetics, transport and mobility, and microenvironmental interactions, providing a foundation for rational co‐design of structure, motion, and reactivity beyond the limits of conventional nanozyme systems. Beyond metal oxide platforms, expanding nanozyme robotics to encompass carbon‐based and MOF‐derived nanozymes will likely require modular architectures that decouple catalytic function from actuation. Incorporating magnetically or optically responsive components as dedicated control modules could broaden material diversity while preserving robotic controllability and supporting translational feasibility.

**FIGURE 6:**
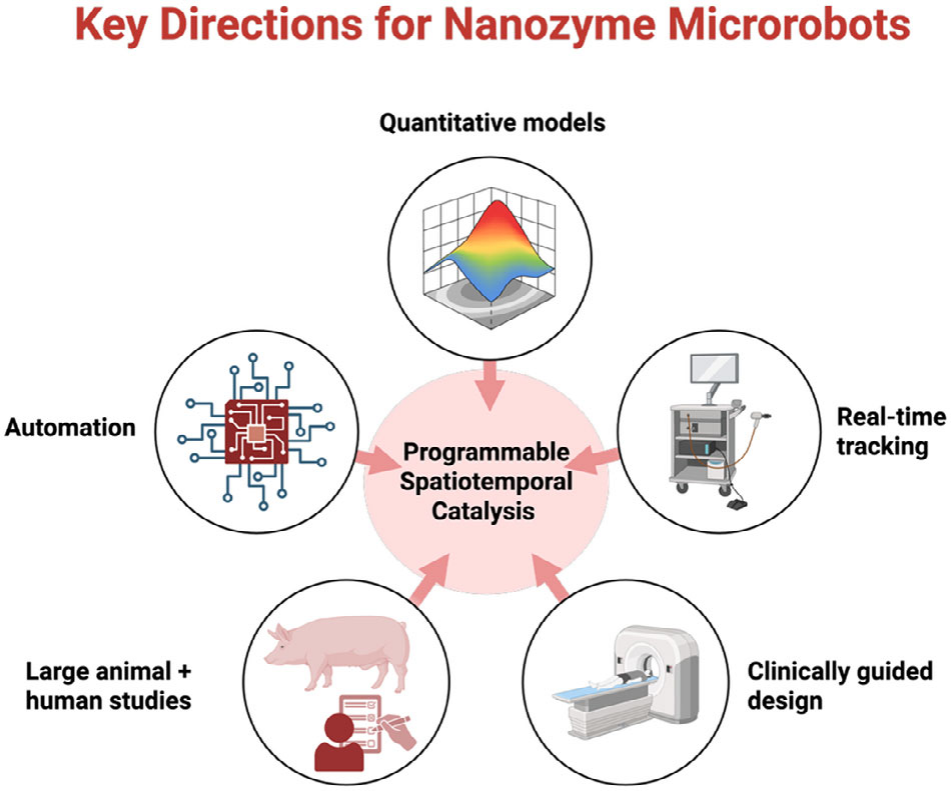
Key directions toward advancing the potential of nanozyme microrobots for precision medicine.

Looking ahead, these design principles provide a blueprint for next‐generation nanozyme microrobots with increased autonomy, multimodal activation, and targeted intervention in complex biomedical settings (Figure 6). Integrating feedback‐controlled actuation with real‐time sensing could further couple locomotion to catalysis, supporting progress toward autonomous, adaptive, and precisely controlled chemical systems. Computational modeling and machine learning are increasingly being applied to optimize performance, adaptability, and intelligent control [85, 86], including closed‐loop adaptive navigation and feedback‐regulated catalysis in constrained environments.

Ex vivo and in vivo demonstrations provide a foundation for clinical translation. Next‐generation systems must balance biocompatibility, retrievability, and multifunctionality, ensuring that catalysis, mobility, and actuation remain effective yet safe in physiological environments. Integration with clinical imaging modalities such as endoscopy, CT, or MRI will be essential for real‐time tracking and guidance. Architectures that enable self‐supplied fuels, ultrasound or optical actuation, and adaptive control could support programmable activation across diverse biological conditions. Together, these advances point toward clinically viable microrobotic therapeutics in which catalysis, motion, and sensing converge to achieve minimally invasive, site‐specific interventions.

Despite notable progress, several challenges must be resolved to enable broad clinical and commercial translation. Catalytic specificity remains a central hurdle, as many nanozymes promote relatively non‐selective redox reactions that may induce off‐target oxidative damage in vivo. Biodegradability and clearance are also unresolved for some inorganic platforms, particularly metal‐containing systems that can persist or accumulate and raise long‐term toxicity concerns. Scalability and reproducibility remain limiting factors, with batch‐to‐batch variation in physical structure and catalytic activity still common. Clinically, progress is further constrained by the lack of standardized evaluation frameworks that quantitatively link physicochemical parameters to catalytic performance and therapeutic outcomes. Addressing these challenges will be a defining milestone for the field and advance nanozyme microrobots for real‐world clinical applications.

## Funding

This research was supported by National Institute for Dental and Craniofacial Research grants: R01 DE025848, R01 DE031491, and R56 DE029985 and by National Science Foundation grant: 2500339.

## Conflicts of Interest

The authors declare no conflicts of interest.

## Data Availability

No new data generated from this work.
